# Case Report: Aumolertinib response as a diagnostic clue for intrapulmonary metastases

**DOI:** 10.3389/fonc.2026.1894879

**Published:** 2026-08-14

**Authors:** Ying Xiao, An Xie

**Affiliations:** Department of Radiology, The First Affiliated Hospital of Hunan Normal University (Hunan Provincial People’s Hospital), Changsha, Hunan, China

**Keywords:** aumolertinib, case report, intrapulmonary metastases, lung adenocarcinoma, next-generation sequencing, treatment response

## Abstract

Lung adenocarcinoma, which is most common in non-smokers and women, rarely presents with diffuse pulmonary nodules. Distinguishing monoclonal intrapulmonary metastases (IPM) from polyclonal multiple primary lung cancers (MPLC) is essential for treatment planning. Although next-generation sequencing (NGS) accurately determines clonal origin, it can be costly and time-consuming. We report a lung adenocarcinoma case with diffuse solid nodules in a 52-year-old non-smoking female. Empirical treatment with aumolertinib resulted in rapid, homogeneous regression of lesions within four weeks, supporting an IPM diagnosis. This uniform treatment response suggests a practical diagnostic indicator for monoclonal disease when NGS is unavailable, facilitating concurrent diagnosis and therapy.

## Background

Lung cancer is the leading cause of cancer-related deaths worldwide, accounting for approximately 18% of all cancer mortality. Among its subtypes, lung adenocarcinoma is the most common in non-smokers and women, typically originating in the peripheral regions of the lungs (1). On chest computed tomography (CT), lung adenocarcinoma usually presents as ground-glass, part-solid, or solid nodules, while a diffuse miliary-like pattern is rare and diagnostically challenging.^2^ Thus the primary clinical task is to differentiate between monoclonal-origin intrapulmonary metastases (IPM) and polyclonal multiple primary lung cancers (MPLC). Next-generation sequencing (NGS) has become the reference method for differentiating between IPM and MPLC by evaluating clonal relationships among lesions. However, high cost, long turnaround time and technical complexity limit its widespread use (2), particularly in patients with multifocal or diffuse lung lesions. We report a case of diffuse lung adenocarcinoma in a 52-year-old non-smoker where empirical aumolertinib treatment without NGS testing resulted in rapid, homogeneous lesion regression within 4 weeks, supporting IPM diagnosis. This suggests treatment response can serve as a valuable diagnostic indicator when NGS is unavailable, enabling simultaneous diagnosis and targeted therapy.

## Case presentation

**Figure 1:**
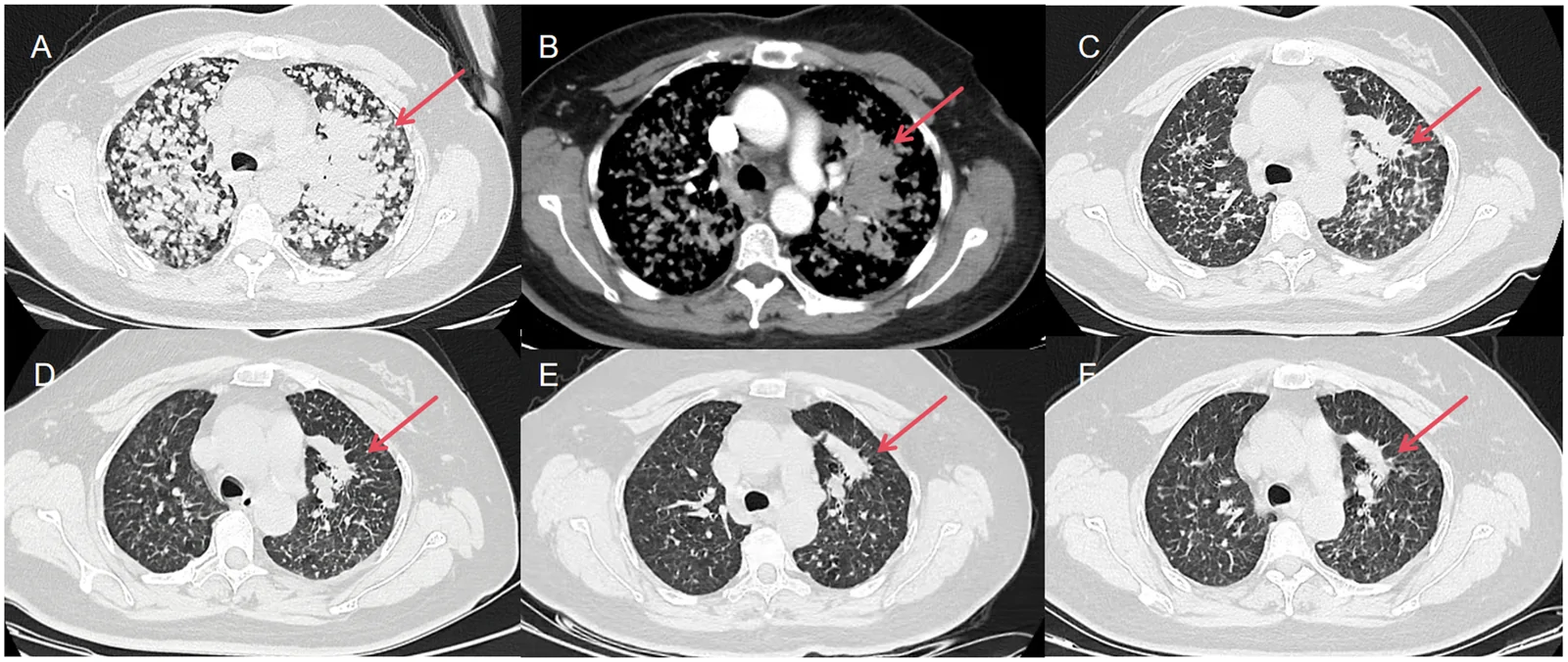
Chronological chest CT evaluation before and after aumolertinib treatment. **(A, B)** Baseline evaluation: **(A)** Lung window image showing diffuse bilateral solid nodules with a dominant lesion in the left upper lung (red arrows, 57 mm); **(B)** Contrast-enhanced mediastinal window image demonstrating mild enhancement of the dominant lesion and the absence of significant mediastinal or hilar lymphadenopathy. **(C–F)** Follow-up lung windows demonstrating therapeutic response over time: **(C)** 1st Assessment (PR): Dominant lesion: 33 mm **(D)** 2nd Assessment (PR): Dominant lesion: 29 mm; lymphangitic improvement; **(E)** 3rd Assessment (PR): Dominant lesion: 28 mm **(F)** 4th Assessment (PR): Dominant lesion: 28 mm.

**Figure 2:**
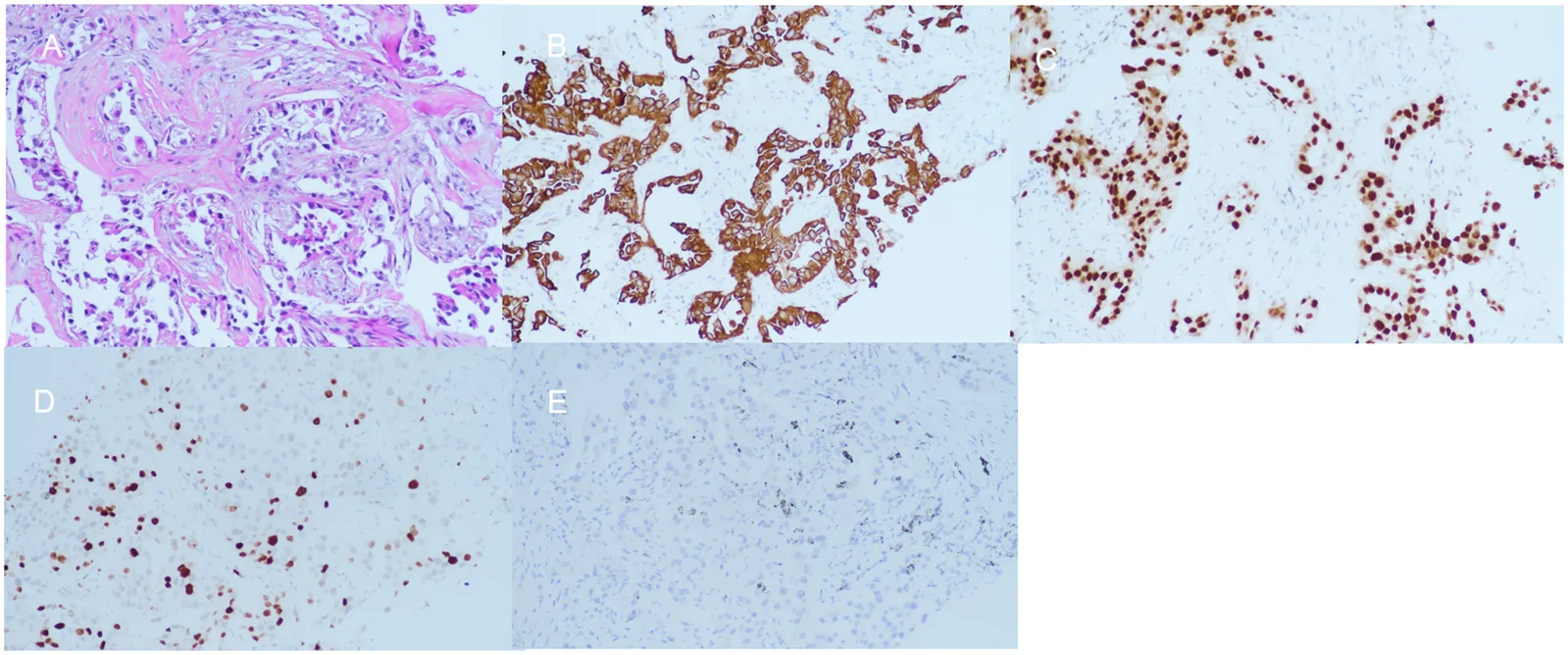
Histopathological and immunohistochemical features of the dominant lung lesion. **(A)** Glandular arrangement of tumor cells (H&E, original magnification ×200). **(B)** Tumor cells showing positive expression for CK7 (IHC, ×200). **(C)** Tumor cells showing positive expression for TTF-1 (IHC, ×200). **(D)** Ki-67 proliferation index is approximately 30% (IHC, ×200). **(E)** Tumor cells showing negative expression for P40 (IHC, ×200).

**Figure 3:**
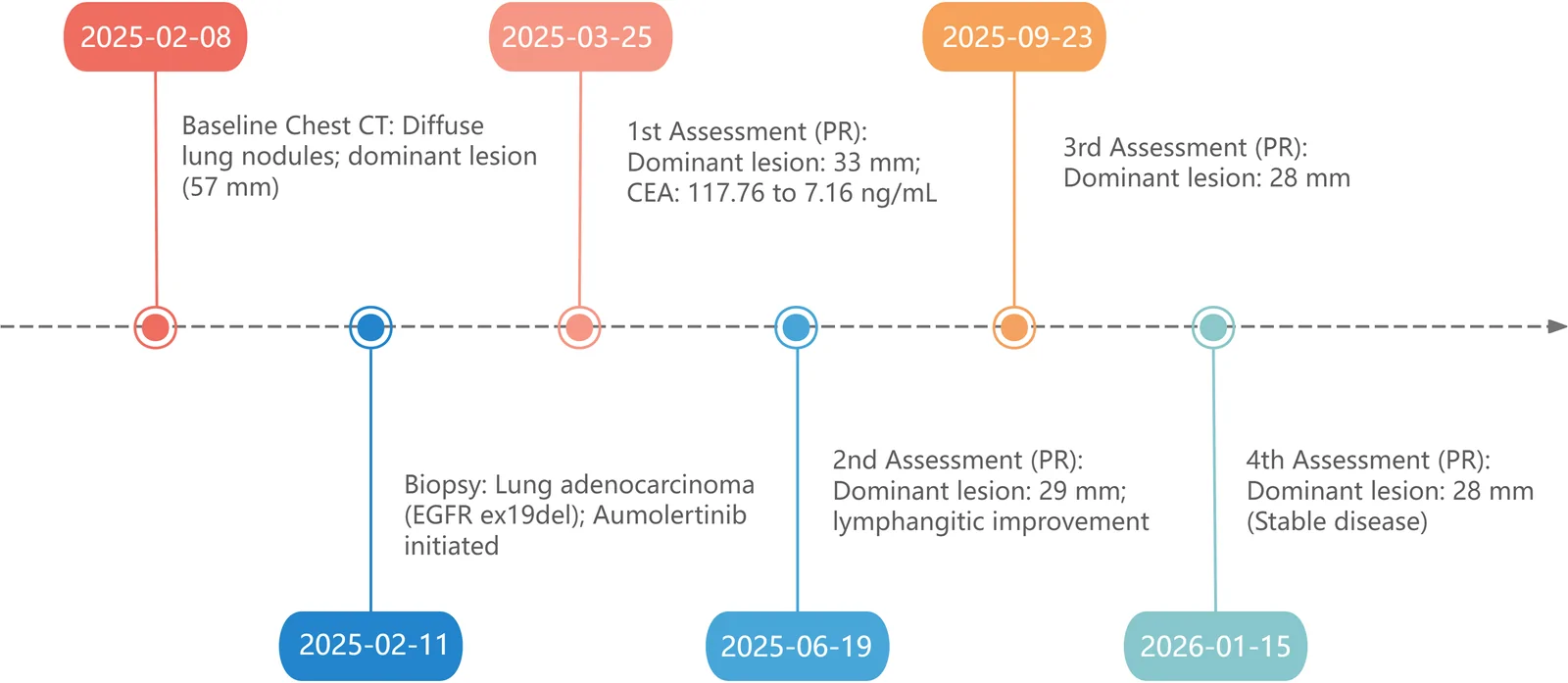
Timeline of aumolertinib treatment and imaging response.

A 52-year-old non-smoking female presented with a 20-day history of dyspnea and cough. Chest CT showed diffuse solid nodules with partial confluence in both lungs, which exhibited mild enhancement on contrast-enhanced images and the largest lesion measured approximately 57 mm in diameter. No mediastinal or hilar lymphadenopathy was observed (**Figures 1A, B**). The findings initially suggested pulmonary tuberculosis or metastases. A comprehensive work-up was performed to exclude extrapulmonary malignancies and infections. Contrast-enhanced abdominal CT, brain MRI, and SPECT bone scintigraphy showed no extrapulmonary primary tumors or skeletal metastases. Multiple sputum smears for acid-fast bacilli and a serum cryptococcal antigen test were negative. Notably, laboratory tests revealed an elevated carcinoembryonic antigen (CEA) level of 117.76 ng/mL (reference < 5 ng/mL). To establish a histopathological diagnosis, a CT-guided percutaneous core needle biopsy was performed on the dominant left upper lesion (**Figure 1A**, red arrow). In diffuse lung disease, multi-site sampling is rarely feasible; instead, a targeted single-lesion strategy is preferred to balance diagnostic yield and procedural safety. Recent studies support such targeted, minimally invasive approaches to optimize diagnostic efficiency in complex thoracic malignancies (3). Pathological examination confirmed lung adenocarcinoma. Immunohistochemical (IHC) staining revealed that the tumor cells were positive for CK7 and TTF-1, negative for P40, and exhibited a Ki-67 proliferation index of approximately 30% (**Figures 2A–E**). The patient was clinically staged with cT4N0M1a lung adenocarcinoma. To guide therapy, targeted next-generation sequencing (NGS) was performed only on the dominant left upper lung lesion (60% tumor content), revealing an *EGFR* exon 19 non-frameshift deletion (p.L747_P753delinsS; MAF: 19.08%). No other actionable somatic alterations were detected; the tumor was microsatellite stable (MSS) and harbored a heterozygous *BCL2L11* polymorphism. Due to diffuse bilateral lung involvement and clinical constraints, multi-lesion molecular analysis was not performed. First-line treatment with aumolertinib (110 mg once daily) was initiated based on EGFR exon 19 deletion in the primary lesion. Aumolertinib was chosen over first-generation EGFR-TKIs due to its guideline-preferred status, favorable efficacy and safety profile, and was preferred to osimertinib based on local availability and institutional experience. The patient had an ECOG performance status of 1 with no significant comorbidities. The overall diagnostic and treatment timeline is summarized in **Figure 3**. Baseline CT on February 8, 2025 identified a dominant lesion in the left upper lung, designated as the target lesion per RECIST 1.1 (SOD: 57 mm). After one treatment cycle (March 25, 2025), follow-up CT demonstrated target lesion shrinkage to 33 mm—a 42.1% reduction—with marked regression of all non-target lesions and no new disease, confirming a Partial Response (PR) (**Figure 1C**). Concurrently, CEA decreased from 117.76 to 7.16 ng/mL, and clinical symptoms improved significantly. Subsequent serial evaluations on June 19 and September 23, 2025, showed continued target lesion regression to 29 mm and 28 mm, respectively, with ongoing resolution of lymphangitic carcinomatosis (**Figures 1D, E**). The latest evaluation on January 15, 2026 showed a stable lesion size of 28 mm (**Figure 1F**). Aumolertinib therapy remains well-tolerated with no severe adverse events, and regular surveillance continues every 2–3 months.

## Discussion

Multifocal lung adenocarcinoma presenting with diffuse, miliary, or lymphangitic carcinomatosis poses a significant diagnostic dilemma in distinguishing MPLC from IPM. This distinction carries important clinical implications, as it directly influences staging, prognosis, and treatment strategies. According to the criteria proposed by the IASLC and subsequent genomic studies, MPLC is often characterized by independent tumor origins and divergent genomic profiles, whereas IPM reflects clonal dissemination from a single primary tumor (4). Recent advances in NGS have significantly improved the ability to assess clonal relationships across multiple lesions, with shared driver mutations strongly supporting a metastatic origin (5). However, in routine clinical practice, especially in patients with multifocal or diffuse pulmonary involvement, definitive classification is often limited by sampling constraints and tumor heterogeneity.

Accumulating evidence suggests that heterogeneous therapeutic responses among multiple pulmonary lesions may reflect underlying intertumoral heterogeneity and distinct clonal evolution, thereby providing clinically relevant clues to tumor relatedness. For instance, heterogeneous treatment responses among multiple pulmonary lesions have been reported in multifocal lung cancer, reflecting potential intertumoral heterogeneity (6–8). Conversely, uniform and rapid responses to EGFR-TKI therapy can be expected in tumors harboring a common EGFR driver alteration, which may indirectly suggest a shared clonal origin. While previous reports have explored treatment response as a complementary indicator of tumor biology, this remains an indirect and inferential marker and cannot replace comprehensive genomic profiling for definitive clonality assessment. Such a “therapeutic diagnosis” approach should be interpreted with caution, as similar radiologic patterns may also arise from biologically independent synchronous lesions that are equally sensitive to targeted therapy, or from lesions sharing common actionable mutations without necessarily confirming a metastatic relationship (9).

## Conclusion

In the present case, the diffuse miliary-like distribution and bilateral pulmonary involvement precluded multi-lesion sampling. The remarkably homogeneous response to EGFR-TKI therapy across all lesions provides supportive clinical evidence favoring a shared EGFR-driven biology and intrapulmonary metastatic spread, while acknowledging the inherent limitations of this inference. This case highlights the potential clinical value of integrating radiologic response patterns with molecular findings in complex multifocal lung cancer, particularly when comprehensive genomic comparison across lesions is not achievable in real-world settings.

## Data Availability

The original contributions presented in the study are included in the article/supplementary material. Further inquiries can be directed to the corresponding author.

## References

[1] Sung H Ferlay J Siegel RL Laversanne M Soerjomataram I Jemal A . Global cancer statistics 2020: GLOBOCAN estimates of incidence and mortality worldwide for 36 cancers in 185 countries. CA Cancer J Clin. (2021) 71(3):209–249. doi: 10.3322/caac.21660 33538338

[2] Dong H Tian Y Xin S Jiang S Guo Y Wan Z . Diagnosis and management of multiple primary lung cancer. Front Oncol. (2024) 14:1392969. doi: 10.3389/fonc.2024.1392969 39411141 PMC11473257

[3] Wang K Hu X Chen Y Yi X Han X Zhu D . Study on the application of percutaneous closed pleural brushing combined with cell block technique in the diagnosis of Malignant pleural effusion. Clin Respir J. (2024) 18:e13705. doi: 10.1111/crj.13705 37775991 PMC10807626

[4] Chou TY Dacic S Wistuba I Beasley MB Berezowska S Chang YC . Differentiating separate primary lung adenocarcinomas from intrapulmonary metastases with emphasis on pathological and molecular considerations: recommendations from the international association for the study of lung cancer pathology committee. J Thorac Oncol. (2025) 20:311–30. doi: 10.1016/j.jtho.2024.11.016 39579981

[5] Yang CY Yeh YC Wang LC Lin YY Lin SY Wang SY . Genomic profiling with large-scale next-generation sequencing panels distinguishes separate primary lung adenocarcinomas from intrapulmonary metastases. Mod Pathol. (2023) 36:100047. doi: 10.1016/j.modpat.2022.100047 36788096

[6] Davis A Jarrar S Ciunci C . Synchronous primary lung cancers containing discrete driver mutations in a never-smoker: Case report. Case Rep Oncol. (2023) 16:1384. doi: 10.1159/000533892 38028577 PMC10645435

[7] Liang N Bing Z Wang Y Liu X Guo C Cao L . Clinical implications of EGFR-associated MAPK/ERK pathway in multiple primary lung cancer. Clin Transl Med. (2022) 12:e847. doi: 10.1002/ctm2.847 35538869 PMC9091990

[8] Lu K Husain H . Intratumoral and intertumoral heterogeneity drives EGFR treatment considerations. J Thorac Dis. (2022) 14:1299–301. doi: 10.21037/jtd-22-312 35693600 PMC9186249

[9] Fukui D Morinaga D Sakakibara-Konishi J Yoshida Y Kashima M Ito S . A case of synchronous multiple primary lung cancers each harboring an EGFR mutation or an ALK fusion gene alone that responded to osimertinib with chemotherapy. Respir Invest. (2026) 64:101375. doi: 10.1016/j.resinv.2026.101375 41564844

